# Macrophage activation syndrome in a 13-month-old child with systemic juvenile idiopathic arthritis mimicking sepsis: a diagnostic challenge and case report

**DOI:** 10.1097/MS9.0000000000005400

**Published:** 2026-07-29

**Authors:** Prasanna Subedi, Shreya Rai, Rashmi Aryal, Anupam Raj Nepal, Pradeep Khatiwada

**Affiliations:** a Department of Internal Medicine, BP Koirala Institute of Health Sciences (BPKIHS), Dharan, Sunsari, Nepal; b Department of Pediatrics, East-West Medical College & Hospital, Dhaka, Bangladesh; c Department of Pediatrics, BP Koirala Institute of Health Sciences (BPKIHS), Dharan, Sunsari, Nepal; d Department of Pediatrics, Kanti Children Hospital, Dharan, Sunsari, Nepal

**Keywords:** case report, cytokine storm, hyperferritinemia, macrophage activation syndrome, secondary hemophagocytic lymphohistiocytosis, systemic juvenile idiopathic arthritis

## Abstract

**Introduction:**

Macrophage activation syndrome (MAS) is a rare but life-threatening hyperinflammatory complication of systemic juvenile idiopathic arthritis (sJIA). Early diagnosis is challenging because MAS frequently mimics severe sepsis, particularly in young children. We report a rare case of MAS in a 13-month-old child with sJIA who presented with severe hyperferritinemia and coagulopathy without hemophagocytosis on bone marrow examination.

**Case presentation:**

A 13-month-old male with sJIA presented with high-grade fever, rash, lethargy, hepatosplenomegaly, and left knee arthritis 2 months after corticosteroid discontinuation. Initial investigations showed pancytopenia and elevated inflammatory markers, and he was treated empirically for sepsis. Persistent fever despite antibiotics together with worsening transaminitis, coagulopathy, hypofibrinogenemia, markedly elevated D-dimer, and hyperferritinemia (7442 ng/mL) raised suspicion of MAS. Blood cultures were negative. Bone marrow examination showed no hemophagocytosis. MAS secondary to sJIA was diagnosed using the 2016 ACR/EULAR criteria. High-dose intravenous methylprednisolone led to rapid clinical and laboratory improvement. The patient remained asymptomatic without relapse at 3-month follow-up.

**Clinical discussion:**

MAS in children younger than 2 years with sJIA is exceedingly rare. This case highlights the diagnostic challenge caused by the overlap between MAS and sepsis. Progressive coagulopathy, rising ferritin levels, and poor response to antibiotics were important clues favoring MAS. The absence of hemophagocytosis did not exclude the diagnosis.

**Conclusion:**

MAS should be considered in children with sJIA presenting with persistent fever, cytopenias, coagulopathy, and hyperferritinemia despite antimicrobial therapy. Early recognition and prompt corticosteroid treatment are critical and may be life-saving, even without bone marrow confirmation.

## Introduction

Systemic juvenile idiopathic arthritis (sJIA) is a rare autoinflammatory disorder of childhood characterized by daily spiking fevers, an evanescent rash, arthritis, and systemic manifestations such as hepatosplenomegaly, lymphadenopathy, and serositis. A potentially life-threatening complication of sJIA is macrophage activation syndrome (MAS). It is a severe form of secondary hemophagocytic lymphohistiocytosis (HLH) caused by excessive immune activation and an uncontrolled cytokine storm^[^1^]^. Here, we report a unique case of MAS in a 13-month-old child with sJIA who presented with severe hyperferritinemia and coagulopathy mimicking sepsis in the absence of hemophagocytosis on bone marrow examination, highlighting the critical importance of early clinical suspicion and prompt initiation of immunosuppressive therapy. Relevant literature was identified through targeted PubMed searches focusing on pediatric MAS associated with sJIA, particularly infantile presentations, sepsis-like manifestations, steroid withdrawal-associated MAS, and cases without hemophagocytosis.

HIGHLIGHTS

- Macrophage activation syndrome (MAS) can closely mimic sepsis in children with systemic juvenile idiopathic arthritis.
- Severe hyperferritinemia and coagulopathy are key laboratory clues for the early recognition of MAS.
- The absence of hemophagocytosis on bone marrow examination does not exclude MAS.
- Prompt initiation of high-dose corticosteroids can be life-saving in suspected MAS.

A key immunopathological feature of MAS is dysregulated activation of T lymphocytes and macrophages, leading to excessive cytokine release with elevated interleukin-1 (IL-1), IL-6, IL-12, IL-18, interferon-gamma (IFN-γ), and tumor necrosis factor-alpha (TNF-α)^[^2^]^.

MAS develops in approximately 10–20% of patients with sJIA and is often triggered by infections, medication changes, or withdrawal of immunosuppressive therapy^[^3^]^. The mean age of onset among children with rheumatic diseases is 4.9 years^[^4^]^, and MAS is exceedingly rare in children younger than 2 years, with only isolated reports^[^5^]^. Early clinical manifestations frequently overlap with severe infection or disease flare, including fever, cytopenias, transaminitis, and coagulopathy, making the diagnosis challenging^[^1,6^]^. Laboratory abnormalities such as hyperferritinemia, hypofibrinogenemia, elevated transaminases, and increased D-dimer can help differentiate MAS from sepsis^[^5^]^.

Diagnosis is guided by the 2016 ACR/EULAR criteria: fever with ferritin > 684 ng/mL plus at least two of the following – platelets ≤ 181 × 10^9^/L, AST > 48 U/L, triglycerides > 1.76 mmol/L, or fibrinogen ≤ 3.6 g/L^[^2,3^]^. However, reliance on these criteria alone may miss early cases, particularly in the early stages of the disease, as some patients may not yet exhibit the full laboratory profile. The patient fulfilled these criteria due to ferritin elevation, thrombocytopenia, elevated AST, and low fibrinogen.

This case has been reported in line with CARE^[^7^]^ and TITAN^[^8^]^ checklists.

## Case presentation

A 13-month-old male with known sJIA, previously treated with oral steroids, which were discontinued two months prior, presented with a three-day history of high-grade fever (40°C), erythematous rash, and lethargy. Examination revealed hepatosplenomegaly and arthritis of the left knee.

**Figure 1.**
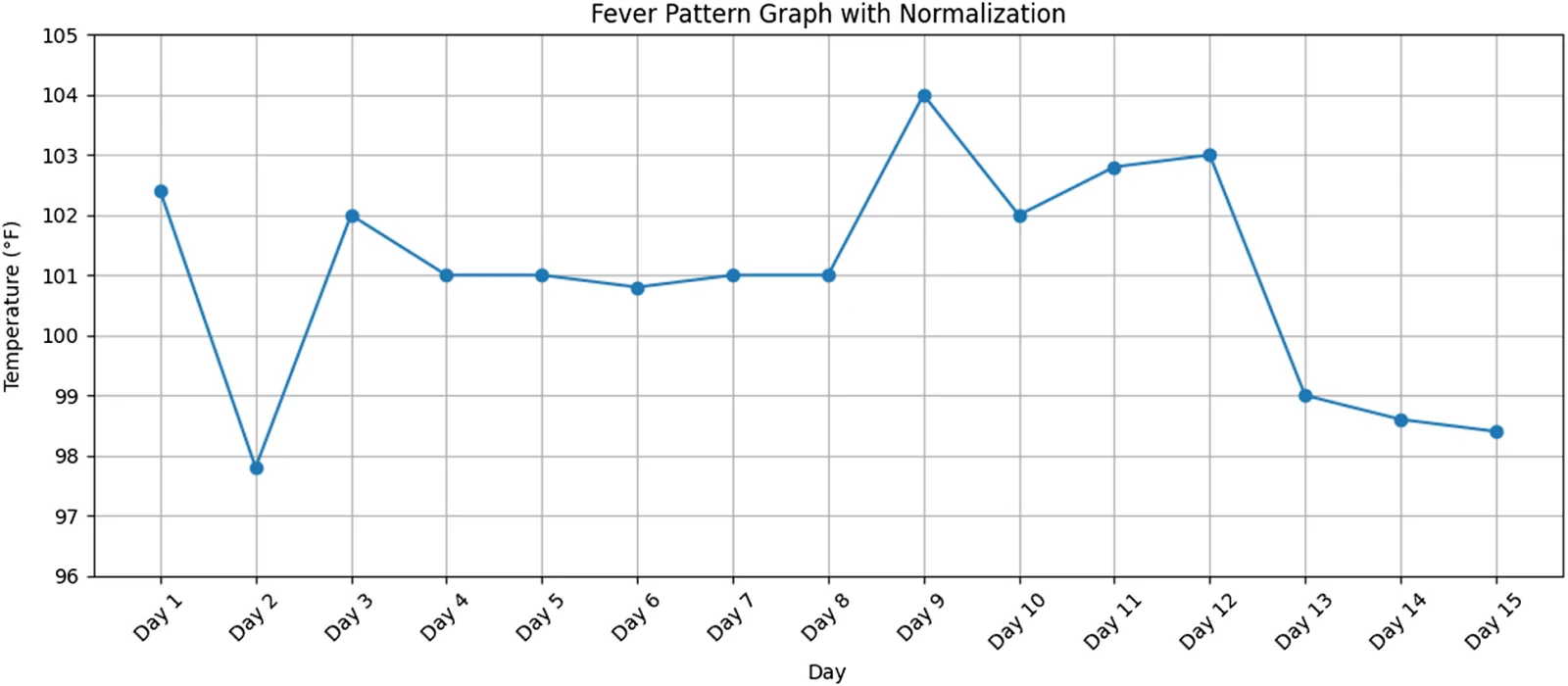
Fever pattern during evaluation and treatment.

Initial labs (Tables 1 and 2) showed pancytopenia (hemoglobin 6.7 g/dL, leukocytes 1.1 × 10^3^/μL, platelets 13 × 10^3^/μL) and an elevated C-reactive protein (138.7 mg/L). With no clear source of infection, broad-spectrum antibiotics were started for presumed sepsis. The fever pattern is shown in Figure 1.

**Table 1:** Laboratory pattern during evaluation and treatment.

Parameter	Day 1	Day 4	Day 6	Day 10	Day 13	Day 15	Normal range
Hb (g/dL)	6.1	7.2	7.2	8.5	7.1	7.7	12–16
TC (/µL)	17 500	6400	6400	1100	6300	14 410	4000–11 000
Neutrophils (%)	67	70	-	57	27	82	40–75
Lymphocytes (%)	25	27	-	36	57	15	20–45
Platelets (/µL)	568 000	473 000	-	133 000	144 000	522 000	150 000–450 000
CRP	134.6	-	129	-	-	-	< 5 mg/L
Reticulocyte (%)	1.2	-	-	-	-	-	0.5–2.5
Urea	13	-	16	23	-	23	10–40 mg/dL
Creatinine	0.33	-	0.32	0.40	-	0.41	0.6–1.2 mg/dL
Na	136	-	139	135	-	137	135–145 mmol/L
K	4.6	-	4.1	4.7	-	4.67	3.5–5.0 mmol/L
AST (SGOT)	35	-	614	1991	158	46	10–40 U/L
ALT (SGPT)	20	-	136	992	392	189	7–56 U/L
ALP	115	-	253	398	256	233	44–147 U/L
Total bilirubin	-	-	0.63	1.18	0.51	0.19	0.2–1.2 mg/dL
Direct bilirubin	-	-	0.10	0.54	0.11	0.02	0–0.3 mg/dL
Albumin	-	-	2.7	3.3	3.06	3.1	3.5–5.0 g/dL
Total protein	-	-	6.2	7.3	6.2	6.01	6.0–8.3 g/dL
PT	12.8	33	14.2	16.2	15	11.5	11–13.5 sec
INR	1.06	2.7	1.18	1.36	1.26	0.94	0.8–1.2
aPTT	48.4	76	63	47	35	26.8	25–35 sec
Ferritin (ng/mL)	-	-	175 993	300 080	-	17 185	13–150
Triglycerides (mg/dL)	-	-	240	717	-	-	< 150
Fibrinogen (mg/dL)	-	-	326	205	-	-	200–400
D-dimer	-	-	2639	7142	-	-	< 500 ng/mL
LDH	-	-	2356	-	-	-	140–280 U/L
Transferrin saturation				26.42%

**Table 2:** Other laboratory findings.

Parameter	Result
Peripheral smear	Microcytic hypochromic
Blood group	A+
Rheumatoid factor	Positive
CSF	Normal
Urine examination	6–8 pus cells, 3–5 epithelial cells/HPF, no RBC
Mantoux	Negative
GeneXpert	Negative
Blood & urine culture	Sterile
Infectious panel	Dengue, Scrub, Malaria, Brucella, Leptospira, Widal, Kala-azar: Negative
Echocardiography	Minimal pericardial effusion
Serum iron	54.89
TIBC	207.76

CSF, cerebrospinal fluid; HPF, high power field; RBC, red blood cell; TIBC, total iron binding capacity.

After 72 hours without improvement and with negative blood cultures, repeat laboratory tests revealed hyperferritinemia (7442 ng/mL), hypofibrinogenemia (205 mg/dL), coagulopathy (PT 33 s, INR 2.7, aPTT 76 s, D-dimer > 5000 ng/mL), and worsening transaminitis (AST 614 U/L, ALT 392 U/L). These findings, along with underlying sJIA and recent steroid withdrawal, suggested MAS.

Bone marrow biopsy on day 6 showed normocellular marrow with eosinophilia (54%) but no hemophagocytosis. Malignancy and infection were excluded. Echocardiography showed mild pericardial effusion, and ultrasound confirmed splenomegaly.

Based on clinical and laboratory findings fulfilling MAS criteria, high-dose intravenous methylprednisolone (30 mg/kg/day) was initiated. The response was rapid, with defervescence within 48 hours and progressive improvement in laboratory parameters. By day 10, platelets rose to 133 × 10^3^/μL, fibrinogen normalized (350 mg/dL), and ferritin decreased to 1200 ng/mL. Coagulation parameters normalized by the second week. The patient was transitioned to oral prednisolone and discharged after 12 days. The patient was afebrile, hemodynamically stable, and eating well at discharge. At the 3-month follow-up, there were no relapses, and the patient was asymptomatic.

## Discussion

MAS has been reported as early as birth in cases of neonatal lupus, but in cases of sJIA it has been reported up to 5 months of age. MAS in sJIA is very rare below 1 year of age and is reported at an average of 4–6 years of age. This case was reported at 13 months of age, which is rare^[^9,10^]^. In previously reported cases, the mean interval between the onset of sJIA and the development of MAS was approximately 5 years (range: 0.75–10 years), indicating that MAS typically occurs later in the disease course^[^11^]^. However, in this case, sJIA was diagnosed at 7 months of age and the interval was only 6 months.

Recognizing MAS in its early stages remains a clinical challenge, particularly because it often mimics common pediatric emergencies such as sepsis. In this case, the child presented with high-grade fever, cytopenias, and markedly elevated inflammatory markers – features shared by both sepsis and MAS. This overlap initially led to treatment for sepsis. However, the lack of clinical improvement despite broad-spectrum antibiotics and adequate coverage raised concerns for an alternative diagnosis^[^1,6^]^.

Laboratory parameters (Table 1) played a pivotal role in shifting suspicion toward MAS. The significantly elevated D-dimer, prolonged PT and aPTT, and low fibrinogen levels suggested a consumptive coagulopathy more typical of MAS than of bacterial infection^[^5,6^]^. These abnormalities, along with rising ferritin and worsening transaminitis, strengthened the clinical suspicion even before a bone marrow evaluation was pursued.

IL-18 plays a central role in MAS pathogenesis by promoting IFN-γ production and a cytokine storm. It is a promising diagnostic and predictive biomarker for MAS in sJIA^[^12^]^, although it was not available in this case.

Although MAS and primary HLH share many clinical and laboratory features, differences in age at onset, triggers, and underlying pathophysiology are important for accurate diagnosis, as treatment and prognosis differ significantly. The MAS/HLH (MH) score uses variables such as age at onset, neutrophil count, fibrinogen, platelet count, and hemoglobin to help differentiate MAS from primary HLH; however, it is only a supportive tool and not diagnostic^[^13,14^]^. High scores (e.g., 148 in our case) may occur in secondary MAS associated with sJIA, infections, or other inflammatory conditions because of substantial overlap in disease features. Therefore, MH scores should always be interpreted in the appropriate clinical context.

The patient’s rapid response to corticosteroids – fever resolution, normalization of counts and coagulation, and declining ferritin – supports the diagnosis and highlights steroid responsiveness.

This rapid improvement supports MAS because corticosteroids quickly suppress the cytokine-driven hyperinflammation characteristic of the disease^[^1,2^]^.

High-dose glucocorticoids remain first-line therapy. Additional treatments, such as cyclosporine, IVIG, or plasma exchange, may be used in refractory cases, though they carry risks such as posterior reversible encephalopathy syndrome^[^14–16^]^. In this case, steroid monotherapy was sufficient.

Compared with previously reported pediatric MAS-sJIA cases, the present case had one of the youngest ages at onset (13 months) and a sepsis-like presentation similar to those in prior reports. Steroid withdrawal was the likely trigger, whereas viral infections were reported in other cases. Bone marrow findings were unremarkable, and early corticosteroid therapy led to a favorable recovery without complications. Unlike prior cases complicated by myocarditis, capillary leak syndrome, recurrent, refractory MAS, or lung disease requiring multiple immunomodulatory agents, this patient responded well to corticosteroid therapy alone (Table 3)^[^17–20^]^.

**Table 3:** Comparison of MAS in sJIA: present case vs previously reported cases.

Parameter	Typical characteristics	Present case	Cortes *et al*	Meneghel *et al*	Slaney *et al*	Prabha *et al*
Age at MAS onset	Typically 3–16 years; rare infant cases reported	13 months	3 years	2 years	2 years	13 years
Sex predominance	Slight female predominance	Male	Female	Male	Male	Male
Interval between sJIA and MAS	Mean ~5 years (range: 0.75–10 years)	6 months	Not mentioned	Dignosed at same episode	11 months	Diagnosed at same episode
Triggering factors (infections like EBV)	Infection, medication changes, disease flare	Steroid withdrawal	RSV infection	Not noted	Adenovirus infection	Not noted
Fever	Persistent high-grade fever (common)	Persistent for weeks	Persistent for weeks	1 months	Persistent	3 months
Hepatosplenomegaly	Frequently present	Present	Not mentioned	Not mentioned	Not mentioned	Present
Lymphadenopathy	Common	Not noted	Reactive lymphadenopathy	Cervical lymphadenopathy	Not mentioned	Present
CNS involvement	Variable (irritability, seizures, coma)	None	None	None	None	None
Anemia	Decreased (anemia common)	Present	Present	Present 8.5 gm/dL	Suggestive of MAS	Present
Platelet count	Decreased (thrombocytopenia typical)		564 000	44 000/mm^3^	Suggestive of MAS	425 000
WBC count	Decreased or normal (cytopenia hallmark)	17 500	36 000	25 990/mm^3^	Suggestive of MAS	14 300
Ferritin	Markedly increased (often > 5000 ng/mL)	175 993	9885 ng/mL	1259	Suggestive of MAS	449 438 pmol/L
Complication	Variable, death	None	None	Acute myocarditis, systemic capillary leak syndrome	Refractory MAS, 5 episodes of MAS	Lung disease
Fibrinogen	Decreased	Decreased	596	1.6 g/L	Not mentioned	3.3 g/L
ESR	Increased	Raised	129	68	Not mentioned	Not Mentioned
Liver enzymes (AST/ALT)	Increased	Raised	23/72	61/45 U/L	1000 U/L	1168/ U/L
Bone marrow findings	Hemophagocytosis (not always present early)	Insignificant	Insignificant	Poor PMN representation, promyelocytes predominant, residual hemophagocytosis	Insignificant	Reactive granulocytic hyperplasia
Clinical presentation	Often mimics sepsis	Sepsis	Sepsis	sJIA	Multifocal pneumonia	Tubercular origin (ATT started)
Time to diagnosis (after admission)	Often delayed	Within a week	1 week	-	Within a week	2 weeks
Treatment	Steroids ± cyclosporine ± biologics	Steroids	Steroid, IVIG, IL6 inhibitor, methotrexate	Steroids, cyclosporine, IVIG, anakinra, ANA, and canakinumab	Steroids, cyclosporine A, anakinra, emapalumab, etoposide, IVIG, IV cidofovir	Steroid, cyclosporine, IVIG
Outcome	Variable; can be fatal if delayed	Improved	Improved	Improved	Improved	Improved

ATT, anti tubercular therapy; EBV, Epstein Bar virus; RSV, respiratory syncytial virus; WBC, white blood cell.

This case is notable for early-onset MAS in a 13-month-old, triggered by steroid withdrawal, presenting with severe coagulopathy and hyperferritinemia without hemophagocytosis, and responding rapidly to corticosteroids.

## Conclusion

MAS in children with sJIA may be clinically indistinguishable from sepsis, particularly in the early phase. Progressive coagulopathy and hyperferritinemia should prompt reconsideration of the diagnosis when fever persists despite adequate antibiotics. In such settings, treatment should not be delayed while awaiting bone marrow confirmation, as high-dose corticosteroids can be life-saving. This case illustrates that bone marrow findings and scoring systems must be interpreted within the clinical context and that early clinical suspicion is crucial.

## Data Availability

Data sharing is not applicable to this article, as no datasets were generated or analyzed during the current study. Data relating to the patient's laboratory investigations can be made available to the editor of the journal on request.

## Research registration unique identifying number (UIN)

Not done.
